# Determinants of consistent condom use among female sex workers in Savannakhet, Lao PDR

**DOI:** 10.1186/s12905-015-0215-0

**Published:** 2015-08-19

**Authors:** Carin Hillerdal Andrews, Elisabeth Faxelid, Vanphanom Sychaerun, Ketkesone Phrasisombath

**Affiliations:** Department of Public Health Sciences, Global Health (IHCAR), Karolinska Institutet, Stockholm, Sweden; Department of Women’s and Children’s Health, Karolinska Institutet, Stockholm, Sweden; Faculty of Postgraduate Studies, University of Health Sciences, Vientiane, PDR Lao; Academic Affair Division, University of Health Sciences, Vientiane, PDR Lao

## Abstract

**Background:**

Female sex workers (FSWs) are a high-risk population for HIV. Correct and consistent use of condoms is the most effective measure for reducing transmission of HIV. Lao PDR is a low HIV-prevalence country, but FSWs have a relatively high HIV prevalence. To be able to make recommendations for condom promotion interventions in Lao PDR it is important to know more about the context specific situation. This study looked at reasons for and associated factors of consistent condom use among FSWs.

**Methods:**

A cross-sectional survey among 258 FSWs in Kaysone Phomvihan district in Savannakhet province was performed.

**Results:**

Almost all FSWs had enough condoms (94 %), condoms always available (100 %) and could always afford condoms (92 %). Consistent condom use was 97% with non-regular partners and 60% with regular partners. Almost all respondents (95 %) had received information about condoms from the drop-in centre. Stated reasons for consistent condom use were prevention of HIV (94 %), STIs (88 %) and pregnancy (87 %). Most reasons for inconsistent condom use were related to partners not wanting to use condoms because of reduced sexual pleasure. Some FSWs reported that they were physically abused and forced not to use condoms. Shorter time in sex work, higher education and FSW not having regular partners were significantly associated with consistent condom use.

**Conclusions:**

Consistent condom use was very high with non-regular partners, but less frequent with regular partners. The main reason for inconsistent condom use was that the partner did not want to use a condom. Associated factors for consistent condom use were not having regular partners, higher education and shorter time in sex work. Condom promotion programs should include both FSWs and their partners and female condoms should be included in condom intervention efforts. Future studies should investigate the validity of self-reported sexual practices, partners’ reasons for inconsistent condom use, risk of violence in sex work and why shorter time in sex work is associated with consistent condom use.

## Background

Sex workers and their clients, men who have sex with men, transgender people, and people who inject drugs are the most affected populations of the HIV epidemic in Asia. One of the major factors in the spread of the epidemic in the region is high HIV prevalence among female sex workers (FSWs) [1]. Stigmatization and marginalisation, limited access to health services, information and means of prevention, and high levels of other sexually transmitted infections (STIs) are factors that put FSWs at an elevated risk of HIV [2, 3]. Prevention programs aimed at FSWs have proven to be among the most effective and they have slowed down the spread of HIV in many countries [2]. Correct and consistent use of condoms is the single most effective measure for reducing transmission of STIs and HIV [4].

Lao People’s Democratic Republic (Lao PDR) is a low-income country in South East Asia with a population of 6.2 million [5]. One hundred percent condom use programs that target FSWs have been introduced with promising results in several South East Asian countries and were also introduced in Lao PDR in 2003 [6]. Even though Lao PDR is classified as a low HIV prevalence country with 0.2% of adults aged 15–49 estimated to be HIV positive, the prevalence is higher among FSWs compared to the general population at between one and four percent [5, 7]. Also STI prevalence among FSWs in Lao PDR is high with 18% for Gonorrhoea and 38% for Chlamydia [7]. These high levels of STIs and HIV in FSWs and the mixing of sexual partners could result in the spread of these infections to large segments of the population [2, 7, 8].

In studies from other settings, relationship intimacy between FSWs and their sexual partners has been found to be an important determinant of condom use, with decreasing use of condoms the more intimate the relationship [9–16]. Other factors contributing to inconsistent condom use are reluctance to ask the partner to use a condom, condom unavailability, older age, drug use, alcohol intake, experiences of violence, financial difficulties, absence of HIV testing and being in a long term relationship [12–15, 17–19]. Conversely good negotiation skills, having a higher income, having children, having access to condoms, being able to afford condoms and confidence in using condoms have been found to increase consistent condom use [12, 13, 15, 16, 19].

No studies have been done on context specific determinants of condom use among FSWs in Lao PDR, even though this information is of high relevance for implementation of the national STI and HIV prevention programmes. The aim of this study was to describe condom use in relation to non-regular and regular sexual partners and reasons for condom use/non-use among FSWs in Lao PDR.

## Methods

### Study setting

Commercial sex work is illegal and perceived negatively, but it is a common feature in the Lao society [20, 21]. Most FSWs are called service women, and they work in entertainment establishments like nightclubs, karaoke bars, restaurants, guesthouses, hotels, and drink shops where they not only sell sex but also serve drinks, converse and dance with guests. In Lao PDR, FSWs are controlled by “mamasans” (pimps) that in exchange provide business arrangements and accommodation to the women. There are a few street-based FSWs in Vientiane, the capital of Lao PDR, and a small proportion of FSWs who clients contact by telephone [22].

Savannakhet province is located 550 km south of Vientiane. It is the most populated province in the country with approximately 826,000 inhabitants [23]. There are 15 districts in Savannakhet province. This study was conducted in Kaysone Phomvihan, the main district of the province, where there are many entertainment establishments where FSWs live and work. Savannakhet has relatively high HIV prevalence with over three percent of FSWs estimated to be HIV positive. STIs are common with Chlamydia and Gonorrhoea rates at 15 and 20% respectively [7]. In a study from 2012, 76% of FSWs reported current symptoms of reproductive tract infections (RTI) including STIs [20].

A drop-in health centre primarily aimed at high-risk populations such as FSWs was established in Kaysone Phomvihan in 2006. The centre provides condoms, STI and HIV testing, and RTI/STI treatment services free of charge. Furthermore, the centre has outreach programs with peer-educators who advocate for condom use and behaviour change [24]. The bar-owners/mamasan/pimps in Kaysone Phomvihan are encouraged to participate in these programs in order to provide health information and encourage FSWs to have regular check-ups for STIs [20].

### Design and participants

A cross-sectional study using closed and open-ended questions was carried out in Kaysone Phomvihan district. The study participants were women who worked in entertainment establishments. The inclusion criteria were women who were able to communicate in Laotian, were willing to participate in the study, and self-reported selling sex.

### Measures

A regular partner was defined as a paying or non-paying partner who the respondent had sex with the week before the interview, and on more than one occasion before this week. The partners who the FSWs had sex with only once were defined as non-regular partners. In the questionnaire the FSW was asked “How frequent did you use condoms over the last 30 days?” This question was asked both regarding regular partners and non-regular partners. If the FSW responded that condom was used every time over the last 30 days with both regular and non-regular partners this was defined as consistent condom use with all partners.

### Data collection and procedures

The study was carried out in March 2012. The aim was to include all FSWs in the district and therefore all existing entertainment establishments in Kaysone Phomvihan were visited. In cooperation with the drop-in centre and local informants, a mapping was done and 276 FSWs from the 35 entertainment establishments were identified as currently working in the district. At the time of the interviews three women declined to participate and another 15 women were absent without contact information. In total 258 FSWs were interviewed, which was a response rate of 94%.

The research team consisted of two medical doctors from Lao PDR, two Swedish master students of Global Health, and one Laotian social scientist. A questionnaire from Family Health International [25] was modified according to the aim of the study and the regional context. The questionnaire included: socio demographic characteristics, access, availability, and affordability of condoms, and condom practices in regard to different types of sexual partners. A Laotian PhD student from Karolinska Institutet translated the questionnaire from English to Laotian and a Laotian translator translated the open-ended replies from Laotian to English.

In order to test the questionnaire and the interviewing procedure, a pilot study was conducted with FSWs during February 2012 in Vientiane. The questionnaire was revised after the pilot study in discussion with the research team members. Before the pilot study we had three different partner definitions in the questionnaire “regular paying client”, “non-paying regular partner” and “non-regular partner”. During the pilot study the FSW could not understand the difference between a “regular paying client” and a “non-paying regular partner”. Many of the FSWs had concurrent long time regular clients who they referred to as “boyfriends” or “husbands” who supported the FSWs in different ways, financially or through other services. In discussion with the research team and the data collectors we therefore decided to use the term regular partner for a paying or non-paying partner that the respondent had sex with the week before the interview, and on more than one occasion before this week.

A two-day training course on the aim of the study, the questionnaire, how to approach FSWs, data collection procedures, and ethical issues was conducted for the data collectors, the coordinator and the translator. The questionnaire was administered by two female data collectors in face-to-face interviews in Laotian in private settings at the FSWs workplaces. Each interview lasted between 15–40 min.

### Data analysis

Firstly the responses from the open-ended questions regarding reasons for consistent and inconsistent condom use with different types of sexual partners were translated from Laotian to English and entered in an excel spread sheet and presented in results under “reasons for inconsistent condom use”. Secondly the data from the closed questions were double entered using EpiData 3.0 (EpiData Association Denmark) and consistency checks were run to explore and compare the two data sets. The original forms were compared with the data sets and incorrect entries were corrected. Data were analysed using STATA v.10 (STATA Corp 2002; College Station, Texas, USA) and descriptive statistics were employed to describe the study population. Finally a logistic regression was performed on associated factors of condom use. The logistic regression was undertaken to further assess the correlates of consistent condom use and to control for confounders. Consistent condom use with all sexual partners during the previous 30 days was treated as the dependent variable and age, time in sex work, having regular partners, education level, marital status, having children, number of sexual partners, having received information on condoms from the drop-in centre, if condoms were received for free, affordability of condoms and having enough condoms were treated as the independent variables. Having non-regular partners and always having available condoms were excluded due to very few negative responses. Bivariate logistic regression was first applied and the independent variables that had a p-value of <0.2 in the bivariate analysis were included in a multiple logistic regression analysis. Independent variables with p-value <0.05 in the multiple logistic regression were considered significant and presented in crude odds ratios and 95% confidence interval.

### Ethical considerations

Ethical approval was received from the Ethics Committee for Health Research at the University of Health Sciences in Lao PDR. Permission to conduct the study was obtained from the local authority in Kayson Phomvihan district. A general permission to interview the FSWs were sought from the bar owner or “mamasan” who provide business arrangements for the FSWs, and are the guardians of the FSWs in their sex work. Some of the FSWs were below 18 years of age, but since obtaining permission from parents would disclose the nature of the daughter´s work, we decided to seek permission from the bar owner or “mamasan” and not from parents. The interviewer explained the study objective and assured participant confidentiality and voluntary participation and then sought verbal consent from each participant before the interview started. Sex work is sensitive and illegal in Lao PDR [21]. In order to prevent participants´ fear for sanctions related to disclosure written consent was thus not sought. The participants received five condoms after the interview.

## Results

### Background characteristics

All of the respondents worked in bars, guesthouses, restaurants or nightclubs. The mean age of the respondents was 21 (range 15–40) and 23% were between 15 and 18 years. About half of the respondents (49 %) had worked as FSWs for less than a year. Only one third (36 %) had completed primary school and a quarter (27 %) had no formal schooling or incomplete primary school. The majority of the respondents (90 %) were not originally from Savannakhet province. More than three quarters (79 %) had never been married and 16% had children. Almost all (95 %) had received information about condoms from the drop-in centre.

### Types of sexual partners

All but one of the respondents reported having non-regular partners the previous week, and 82 (32 %) also had regular partners. The mean number of partners in the previous week was 3 (range 1–25) for non-regular partners and 1.4 (range 1–5) for regular partners.

### Condom practices with non-regular and regular partners

All respondents had experience of using condoms during the last month. Consistent condom use 30 days prior to the survey was 85% with all sexual partners, 97% with non-regular partners and 60% with regular partners. All respondents had used condoms during their last sex with a non-regular partner while 73% had used condoms during their last sex with a regular partner. During the last sex it was common that the respondent herself suggested condom use (91 % with non-regular partners and 62 % with regular partners). Considering the last sex when the partner did not want to use a condom, all respondents said that they had tried to persuade the partner if he was a non-regular partner while 79% said that they had tried to persuade a regular partner (Table 1).

**Table 1:** Condom practices

Variables	*N* = 258		%	
Consistent condom use previous 30 days with all sexual partners				
Yes	218		84.5	
No	40		15.5	
Variables	Non-regular partner		Regular partner	
	*n* = 257	%	*n* = 82	%
Consistent condom use previous 30 days				
Yes	249	96.9	49	59.8
No	8	3.1	33	40.24
Condom use during last sex				
Yes	257	100.0	60	73.2
No	00	00	22	26.8
Suggestion of condom use				
Myself	232	90.3	51	62.2
My partner	2	0.8	2	2.4
Joint decision	23	8.9	13	15.9
Condom use was not suggested	00	0.0	16	19.5
Tried to persuade partner to use a condom				
Yes	256	100.0	65	79.3
No	00	0.0	17	20.7

### Reasons for consistent condom use

The reasons for consistent condom use with non-regular partners were prevention of HIV/AIDS, prevention of other STIs, and prevention of pregnancy. More than two-thirds of the respondents (70 %) stated a combination of preventing HIV/AIDS, other STIs and pregnancy (Table 2).

**Table 2:** Reasons for consistent condom use with non-regular partners in the last 30 days (*n* = 249)

Reasons for consistent condom use^a^	Respondents	
	*n* = 249	%
Prevent HIV/AIDS	233	93.6
Prevent STI	218	87.6
Prevent pregnancy	217	87.2
Answered a combination of prevent HIV/AIDS/STI/pregnancy	173	69.5

^a^Multiple responses were allowed, the sum of the responses are therefore greater than 100 %

### Reasons for inconsistent condom use

Most of the reasons for inconsistent condom use with regular partners were related to the respondent’s partners not wanting to use condoms. The regular partners’ most common reason for not wanting to use condoms was related to reduced sexual pleasure when using condoms. Some of the partners’ reasons were also directly related to difficulties when using condoms: allergic reactions to condoms, difficulties with ejaculation and sustaining erection, and the condom being to small to fit the penis. Inconsistent condom use was also alcohol related with two respondents saying that drunken partners did not want to use condoms. Respondents also reported that their regular partners did not want to use condoms due to the intimacy of their relationship, as shown in the following two quotes from partners; *“If you love me, we should not use condoms”* and *“We should trust each other”*. Some reasons for inconsistent condom use with regular partners were related to the respondents themselves such as; mutual love and/or trust between the respondent and the partner, respondent forgot to use condoms, and reduced sexual pleasure for both partner and respondent.

All but one reason for not using condoms with non-regular partners were related to the partners not wanting to use condoms. The non-regular partners’ major reason for not wanting to use condom was also related to reduced sexual pleasure when using condoms. One respondent said that one of her non-regular partners did not want to use condoms because he had difficulties to stay erect and ejaculate when using condoms. Two of the FSWs also said that the non-use of condom was involuntary and forced by partners through physical abuse, and one of the FSWs said that partners took of the condom during sexual intercourse. The only respondent-related reason was that one of the FSWs said she had forgotten to use condoms.

### Accessibility of condoms

The most frequently used sources for accessing condoms were the drop-in centre (83 %) and peer-educators (15 %). The reasons for choosing these sources were that condoms were free of charge, counselling was provided, condoms were always available, and proximity to the respondent's workplace. The sources where the respondents would prefer to have access to condoms were at the drop-in centre, from mamasan or peer educators, and at the guesthouses (Table 3).

**Table 3:** Accessibility of condoms

Sources of condoms^a^	Most frequently used		Preferred sources	
	*n* = 258	%	*n* = 258	%
Drop-in centre	214	82.9	184	71.3
Peer-educator	38	14.7	93	36.0
Mamasan	18	7.4	104	40.3
Guesthouse	9	3.5	72	27.9
Pharmacy	9	3.5	24	9.3
Reasons for most frequently getting condoms from these sources^a^	*n* = 258		%	
Free	234		90.7	
They give counselling	155		60.1	
They always have condoms	147		57.0	
Close to workplace	62		24.0	
Cheap	24		9.3	

^a^Multiple responses were allowed, the sum of the responses are therefore greater than 100 %

### Affordability and availability of condoms

More than half of the respondents received condoms for free from the source (e.g. drop-in centre) they most frequently used for accessing condoms. Almost all of the respondents reported that they could always afford to buy condoms (92 %) and that they always had condoms available from the sources most frequently used (Table 4).

**Table 4:** Affordability and availability to condoms

Variables	Frequency (*n* = 258)	Percentage (%)
Have enough condoms according to needs		
Yes	243	94.2
No	15	5.8
Get condoms for free from the place where condoms are most often collected		
Yes	149	57.8
No	109	42.2
Can always afford to buy condoms		
Yes	236	91.5
No	22	8.5
Always have available condoms from place where condoms are most often collected		
Yes	257	99.6
No	1	0.4
Number of condoms needed per day; median 2; mean 2.7; range 2-10		

### Association between independent variables and consistent condom use

The independent variables included in the bivariate logistic regression analysis were age, time in sex work, having regular partners, education level, marital status, having children, number of sexual partners last week, having received information on condoms from the drop-in centre, if condoms were received for free, affordability of condoms, and having enough condoms. Having non-regular partners and always having available condoms were excluded due to very few negative responses. The following independent variables, time in sex work (p = 0. 001), education level (p = 0.050) having regular partners (p = 0.000), if condoms were received for free (p = 0.177) and number of sexual partners last week (p = 0.133) had a p-value of <0.2 and were included in the multivariate logistic regression model. The variables that were significantly associated with consistent condom use in the multivariate logistic regression model (p-value of <0.05) were having worked as FSW less than 6 months compared to more than one year (OR: 6.15; 95 % CI: 1.83-20.60); p = 0.003), having a secondary level education or higher compared to primary education or lower (OR: 3.39; 95 % CI: 1.29-8.87; p = 0.013) and not having a regular partner (OR: 21.69; 95 % CI: 7.98-58.93; p = 0.000) (Table 5).

**Table 5:** Association between consistent condom use with all sexual partners previous 30 days, and independent variables using bivariate and multivariate logistic regression (*n* = 258)

Background characteristics	Crude OR^a^ (95 % CI^b^)	P-value	Adjusted OR^a^ (95 % CI^b^)	P-value
Age in years				
25–40	1			
19–24	1.10 (0.48-2.55)	0.823		
15-18	2.54 (0.79-8.16)	0.118		
Marital status				
Not married	1			
Currently married	5.28 (0.69-40.08)	0.108		
Having children				
Have children	1			
Don’t have children	1.11 (0.45-2.71)	0.820		
Duration of sex work				
> 1 year	1		1	
6–11 months	0.77 (0.31-1.92)	0.580	0.85 (0.28-2.59)	0.780
< 6 months	6.13 (2.07-18.12)	0.001	6.15 (1.83-20.60)	0.003
Educational status				
≤ Primary school	1		1	
≥ Secondary school	2.20 (0.99-4.85)	0.050	3.39 (1.29-8.87)	0.013
Number of sexual partners last week				
2–3	1			
4–5	0.64 (0.29-1.36)	0.244	1.02 (0.39-2.68)	0.975
6-25	0.48 (0.18-1.25)	0.133	1.10 (0.34-3.63)	0.871
Having regular partners				
Yes	1		1	
No	20.07 (7.96-50.62)	0.000	21.69 (7.98-58.93)	0.000
Have received information on condoms from Drop in centre				
Yes	1			
No	2.47 (0.31-19.45)	0.390		
Get condoms for free from the place where condoms are most often collected				
Yes	1			
No	1.63 (0.80-3.34)	0.177	0.69 (0.288-1.69)	0.425
Can always afford to buy condoms				
Yes	1			
No	0.81 (0.26-2.53)	0.717		
Have enough condoms according to needs				
Yes	1			
No	0.48 (0.14-1.58)	0.227		

^a^ *OR* odds ratio

^b^95 % *CI* 95 % confidence interval

## Discussion

FSWs in Savannakhet appear to have access to enough and affordable condoms all the time, and their knowledge about the benefits of condom use was high. Most likely this is because health information and condom distribution were accessible to the women from the drop-in centre and through peer-educators outreach activities. Higher education was found to be associated with consistent condom use among FSWs and could be explained through greater exposure to information about risks with not using condoms and being able to read and understand written information.

We found that almost all FSWs in this study used condoms consistently with non-regular partners. But the high rates of HIV and other STIs in the province [7, 20] also indicate that these self-reported rates of consistent condom use might be an overestimate. Self-reported sexual practices are subjected to recall bias and deliberate concealment and studies have shown that there is discordance between self-reported rates of condom use and observed levels of unprotected sex [26, 27]. Future studies should investigate the validity of self-reported sexual practices among FSWs in Lao PDR.

Condom use with non-regular partners was high (97 %) but considerably lower with regular partners (60 %), and not having regular partners was associated with consistent condom use. Since most respondents had several regular partners during the same week (range 1–4) these low rates of condom use with regular partners is a high-risk sexual behaviour. The findings show that the main reason for not using condoms both with regular and non-regular partners was that partners did not want to use condoms. The partners’ reasons were mainly reduced sexual pleasure, difficulties when using condoms, and that condoms should not be used in intimate relationships. It is therefore important to include partners of FSWs in condom promotion interventions, and further research should include FSWs’ partners for a better understanding of how to change their behaviour of not wanting to use condoms.

Although there were few reports of violence, the two FSWs being physically abused by non-regular partners and forced not to use condoms is a clear example of how vulnerable this group is. Violence has been shown in previous studies to be a determinant of inconsistent condom use among FSWs and can result in morbidity, mortality, emotional scarring, and HIV-infection [3, 18]. FSWs in entertainment venues provide sexual services in guesthouses, hotels or in the partner’s room, sometimes these venues are attached to their workplace, but sometimes the FSWs will go with their partners to other settings. In a previous study in this setting, FSWs suggested that bringing a cell phone when going outside the workplace or go to a venue with a security guard who can assist when needed, choosing clients carefully, and doing what client asks for could work as security measures against violence [28]. But the associated risks of violence for FSWs in Lao PDR needs to be further investigated to developed strategies for how violence can be minimised.

One way of empowering FSWs and to address the issue of men not wanting to use condoms, or forcing FSWs to not use condoms is to include female condoms in condom interventions. Female condoms are initiated and controlled by the women and not dependent on male cooperation. FSWs might feel safer using the female condom since they can make sure it has not been tampered with before insertion and partners cannot easily remove the condom during sex. FSWs also report using the female condoms without the partner’s knowledge when they refused to use male condoms [29]. It has also been shown that interventions that promote use of both male and female condoms are more effective compared to those that promote male condoms only [30].

FSWs that had worked less than six months were more likely to use condoms as compared to FSWs that had worked for a year or more. One explanation could be that young FSWs who were new in sex work had more sexual clients compared to their older peers as has been shown in other setting [31]. It has been shown in other studies that FSWs who have worked for many years receive fewer clients and in order to keep clients FSWs often accept sex without a condom [14]. Newcomers might also fear consequences such as STI and pregnancy more than the experienced FSWs. A qualitative study from the same setting suggested that “mamasans” usually recommended their sex workers to use condom in order to avoid contracting STIs as well as to keep the entertainment venues reputation [32]. The “mamasans” might counsel and encourage condom use more when the FSWs are introduced to the sex work, thinking FSWs that have worked longer does not need the same attention. However, why shorter time in sex work is associated with consistent condom use needs to be investigated in future studies since these findings do not resemble other findings from this setting [20].

This study has some methodological considerations. All entertainment establishments that were known in the district were included, but there is a risk of missing hidden locations and high-class as well as highly mobile FSWs. The use of a questionnaire that was partly newly developed and therefore not validated can have influenced the study results negatively. However, using a local coordinator with high knowledge of the area, testing the questionnaire in the pilot study, and using female interviewers were ways to improve the validity.

## Conclusions

Consistent condom use was very high with non-regular partners, but less frequent with regular partners and the main reason for inconsistent condom use was that partners did not want to use condoms. Associated factors for consistent condom use were not having regular partners, higher education and shorter time in sex work. Condom promotion programs should include both FSWs and their partners and female condoms should be included in condom intervention efforts. Future studies should investigate the validity of self-reported sexual practices, partners’ reasons for inconsistent condom use, risk of violence in sex work, and why shorter time in sex work is associated with consistent condom use.
